# Multiplexed Plasmonic Nano-Labeling for Bioimaging of Cytological Stained Samples

**DOI:** 10.3390/cancers13143509

**Published:** 2021-07-13

**Authors:** Paule Marcoux-Valiquette, Cécile Darviot, Lu Wang, Andrée-Anne Grosset, Morteza Hasanzadeh Kafshgari, Mirela Birela, Sergiy Patskovsky, Dominique Trudel, Michel Meunier

**Affiliations:** 1 Department of Engineering Physics, Polytechnique Montréal, Montreal, QC H3T 1J4, Canada; paule.marcoux@hotmail.com (P.M.-V.); cecile.darviot@polymtl.ca (C.D.); lu.wang@polymtl.ca (L.W.); Morteza.kafshgari@polymtl.ca (M.H.K.); sergiy.patskovsky@polymtl.ca (S.P.); 2 Centre de Recherche du Centre Hospitalier de l’Université de Montréal (CRCHUM) et Institut du Cancer de Montréal (ICM), Montréal, QC H3T 1J4, Canada; andree-anne.grosset.chum@ssss.gouv.qc.ca (A.-A.G.); mirela.birlea.chum@ssss.gouv.qc.ca (M.B.); dominique.trudel.chum@ssss.gouv.qc.ca (D.T.); 3 Department of Pathology and Cellular Biology, Université de Montréal, Montreal, QC H3T 1J4, Canada

**Keywords:** cytopathological cancer diagnosis, paraffin-embedded cytological tissues, gold nanoparticles, gold-silver alloy nanoparticles, plasmonic biomarkers, immunoplasmonic-multiplexed-labeling, side-illumination microscopy, bioimaging

## Abstract

**Simple Summary:**

The improvement in the reliability and precision of traditional cytopathological examination protocols (semi-quantitative cancer diagnostics) is a persisting challenge. Many developed high-tech diagnostic approaches have also been declined due to their complexity, non-complementary, and problematic integration with standard pathology laboratory equipment and protocols. In this study, a complementary bioimaging approach based on plasmonic nanoparticles (NPs), due to their stable, strong scattering feature, is therefore developed. This type of approach resists against a strong background of the cytological counterstaining while simultaneously delivering ancillary diagnostic information by using the same cytological stained samples. The direct observation and analyses of four types of plasmonic NPs with different scattering colors on hematoxylin and eosin (H&E) paraffin-embedded specimens are demonstrated. This is performed while using a well-designed adapter for side-illuminated (SI) dark-field conventional microscopy without interfering with traditional cytopathology strategies. This state-of-the-art integrated bioimaging approach (observation of plasmonic NPs on H&E-stained cytology samples) constitutes an indispensable tool that improves not only cancer diagnosis but also daily care.

**Abstract:**

Reliable cytopathological diagnosis requires new methods and approaches for the rapid and accurate determination of all cell types. This is especially important when the number of cells is limited, such as in the cytological samples of fine-needle biopsy. Immunoplasmonic-multiplexed- labeling may be one of the emerging solutions to such problems. However, to be accepted and used by the practicing pathologists, new methods must be compatible and complementary with existing cytopathology approaches where counterstaining is central to the correct interpretation of immunolabeling. In addition, the optical detection and imaging setup for immunoplasmonic-multiplexed-labeling must be implemented on the same cytopathological microscope, not interfere with standard H&E imaging, and operate as a second easy-to-use imaging method. In this article, we present multiplex imaging of four types of nanoplasmonic markers on two types of H&E-stained cytological specimens (formalin-fixed paraffin embedded and non-embedded adherent cancer cells) using a specially designed adapter for SI dark-field microscopy. The obtained results confirm the effectiveness of the proposed optical method for quantitative and multiplex identification of various plasmonic NPs, and the possibility of using immunoplasmonic-multiplexed-labeling for cytopathological diagnostics.

## 1. Introduction

Early diagnosis of cancer plays a critical role in the prevention of morbidity (an estimated 9.6 million deaths, or one in six deaths, in 2018) and global cancer burden [1]. The accuracy of cancer diagnosis and the potential outcome for successful treatment depends on many aspects, including the reliability and precision of existing clinical and pathological examination protocols, as well as the professional level of practicing pathologists [2].

The gold standard protocol in cytopathology involves hematoxylin and eosin (H&E) staining and its ability to clearly define the underlying tissue morphology by staining nuclei and cytoplasm in different colors. It is also used as a control for all immunohistochemical (IHC) stains to show that the tissue has been processed correctly and is free of artifacts [3,4,5,6]. A recent study successfully demonstrated that when IHC is used for the visualization of the expression pattern of proteins (e.g., of cancer cells), H&E counterstaining can be applied in order to facilitate a simultaneous observation of the same stained samples [7]. However, these diagnostic approaches still have some disadvantages and limitations, which should be addressed [8]. The preparation of histo-cytopathological specimens includes treatment with cytological fixatives, and other embedded components that affect the antigenicity of cells, thereby reducing the effectiveness and reliability of immunohistochemistry [9,10]. Although the two-color IHC protocol was developed by labeling two antigens located in different cellular compartments, antigens are usually identified one at a time, making it difficult to confirm the presence of multiple overexpressed receptors in the same cells [11]. The use of fluorescent antibodies and dyes for an accurate antigen detection, due to their photobleaching feature, is the other major drawback that prevents the integration of immunofluorescence in cytopathology [12].

As a result, there is a need for a reliable and preferably multiplexed immunological method for cell identification and differentiation. To address these problems, quantum dots (QDs) have been proposed as alternative multiplexed biomarkers. However, the problems of the QDs’ stability in the intracellular environment, a short shelf life, and the difficulty of detecting fluorescence in stained tissues necessitates a search for another alternative [13,14]. On the contrary, plasmonic nanoparticles (NPs) as stable and biocompatible markers have demonstrated a great potential for biomedical applications [15,16,17,18,19]. Existing technology to fine-tune NPs physicochemical properties (e.g., geometry, size, and composition) allows for the generation of a multitude of NPs with spectrally distinguishable absorption/scattering peaks in the visible or near infrared, thereby enabling multispectral imaging and multiplex immunolabeling [20,21,22]. Moreover, direct observation and analysis of even single plasmonic NPs can be performed using conventional dark-field or backscattering microscopy, which are readily available in research laboratories [10,23].

Many of the latest high-tech medical technologies developed for the diagnosis of cancer have not been accepted by medical professionals in healthcare settings due to their complex and problematic integration with laboratory devices and pathology protocols. To create an opportunity for this innovative biomedical technology’s adoption and utilization, it must pass several steps of development. The initial step toward acceptation relies on the possibility of new bioimaging approaches generating additional analytical information for the same specimens while not interfering with standard pathology protocol. The final step will be a complete replacement of the conventional diagnostic method with the new and more efficient technology.

In this study, we describe a modern biolabeling strategy based on plasmonic NPs (nanoplasmonic markers) with different spectral signatures. As a proof-of-concept, we demonstrated multiplex plasmon bioimaging on H&E, formalin-fixed paraffin-embedded (FFPE) specimens using a specially designed side-illuminated (SI) dark-field adaptor integrated into a conventional microscope and used for analysis of cytopathological specimens. We have comparatively confirmed that direct observation of plasmonic NPs is possible without interfering with traditional strategies of cytopathology diagnostics.

## 2. Materials and Methods

**Materials**: Gill II hematoxylin, Harris hematoxylin, eosin B, formalin, and ethanol, were obtained from Sigma-Aldrich (St. Louis, MO, USA). The eosin Y solution was purchased from Leica Biosystems (Wetzlar, Germany) and diluted 1:2 in ethanol 100% in this study. The phosphate-buffer saline, Gibco Dulbecco’s Modified Eagle Medium (DMEM), Fetal bovine serum (FBS), penicillin-streptomycin-glutamine (100X), and HistoGel™ were obtained from ThermoFisher Scientific (Waltham, MA, USA). All slides were mounted in Sub-X mounting medium (Electron Microscopy Sciences, Hatfield, PA, USA) compatible with xylene substitute (VWR International, Radnor, PA, USA). The gold NPs and nanorods were purchased from Nanopartz, Inc. (Loveland, CO, USA). The other plasmonic NPs (Table 1), including the gold-silver Alloy NPs (50/50 and 10/90 vol.), were synthesized based on a seeded-growth approach (Turkevich method), and their optical properties match the SI microscopy adaptor [10,16,22,24].

**Cytology sample preparation**: Two types of cytology samples were prepared by using the same breast cancer cell line (MDA-MB-453). The cells were cultured in DMEM and supplemented with FBS (10% vol.) and penicillin-streptomycin-glutamine (1% vol.).

The non-embedded cell samples (type-I) were prepared by using adherent cells (three dimensional = 3D) on a microscope slide. These samples could be compared to a cytology smear in which the cells are deposited and dried on a microscope slide. The prepared cells were incubated for 36 h in 8 well-plate (IBIDI, Gräfelfing, Germany) with removable chambers to reach a confluence of ~80%. Afterward, the cells were rinsed with PBS, and the wells were filled with fresh DMEM containing fresh plasmonic NPs (1 × 10^6^ NPs/wells). After incubation at 37 °C, the unbound plasmonic NPs were removed by washing the cells with PBS. The prepared cells were then fixed in formalin (10% vol., 10 min) and the grid was removed.

The preparation of the FFPE samples (type-II) first required the cells to be centrifugated and then fixed with formalin (10% vol.). Afterward, the fixed cells were mixed with HistoGel™ to create a cell pellet, embedded in paraffin, and mounted on a slotted cassette. The sample was cut into thin slices (4 µm thickness), transferred and dried on a positively charged microscope slide (Superfrost PLUS from FisherBrand Lab Equipment, Pittsburgh, PA, USA). The cells were then deparaffinated and rehydrated in xylene substitute, ethanol, and distillated water baths. Afterward, the solution of fresh plasmonic NPs (1 × 10^6^ NPs in DI water) was pipetted on top of the FFPE sample, creating a bubble on the slide, and incubated at room temperature. Before staining, the prepared samples, the excess of NPs was removed by immerging the samples into water baths.

Next, both types of samples were stained with Gill II hematoxylin and eosin B or Y, as well as Harris hematoxylin and eosin B or Y, and then dehydrated by placing the prepared slides in consecutive ethanol (70, 90, and 100% vol.) and xylene substitute baths. Afterward, a drop of Sub-X mounting medium was added, before covering the samples with the glass coverslip.

**Figure 1:**
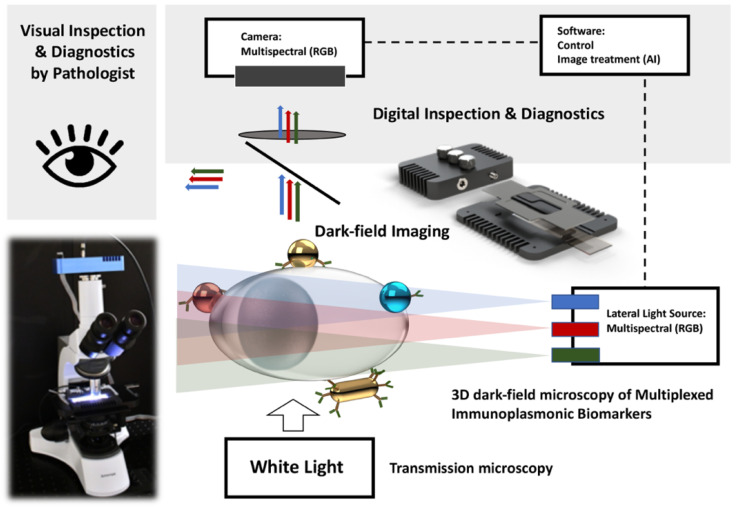
A schematic representation of a multimode microscopy setup integrated with VegaPhoton SI adaptor facilitating the visualization of red, green, blue, and yellow color scattering plasmonic NPs on H&E-stained cytology samples. As illustrated, different plasmonic NPs with their unique resonance peak on single cell are clearly visible in this setup. The multimode microscopy setup is composed of a (I) transmission microscopy (white light source) and (II) 3D dark-field microscopy (SI observation of plasmonic NPs) and can independently visualize each mode by simply switching from one mode to another.

**Microscopy setup with side-illuminated dark-field imaging mode**: To meet the above-mentioned requirements, the design of the optical microscopy system should provide a conventional transmission microscopy mode for bright-field imaging of cytopathological samples in combination with a dark-field imaging mode for the detection of plasmonic markers. In Figure 1, we present a schematic of the proposed optical setup built on a conventional upright microscope, which is commonly used by pathologists.

As known from the literature, various dark-field techniques can be used for the direct observation and imaging of plasmonic NPs. However, in our case, the lateral illumination dark-field method is the most optimal for several reasons. First, the SI dark-field adapter (VegaPhoton Inc., Montreal, QC, Canada) can be easily integrated into the microscope stage by simply replacing the conventional glass slides holder. Secondly, the principle of side illumination is based on the coupling and propagation of light in a waveguide, and a standard 1 mm microscope slide, used in the cytophatology preparation, serves as a waveguide. In addition, the recent development of Red-Green-Blue (RGB) miniature light-emitting diodes (LEDs) allows direct LED light coupling to microscope slides and capability of fast manual or automatic light intensity modulation. The light source is a tricolor LED array for RGB illumination placed on either side of the support that fits a conventional slide (75 mm × 25 mm) [25]. This SI adaptor can be easily installed on any type of bright-field microscope (upright or inverted) and does not require an extra modification to be assembled into microscopes or oil-immersion objectives.

The developed optical setup makes it possible to observe cytological samples with H&E staining in a bright field, and then investigate the labeling with plasmonic NPs in the same place by simply switching from bright-field illumination to the LEDs of the built-in SI adapter. In this work, images in bright-field mode and with the SI adaptor were taken using the Nikon 60× objective (0.7 numerical aperture = NA) with a variable working distance from 1.8 to 2.6 mm and the RGB color sCMOS PCO-Panda 4.2 camera (PCO AG, Kelheim, Germany).

## 3. Results

### 3.1. Side-Illuminated Dark-Field Imaging

**Figure 2:**
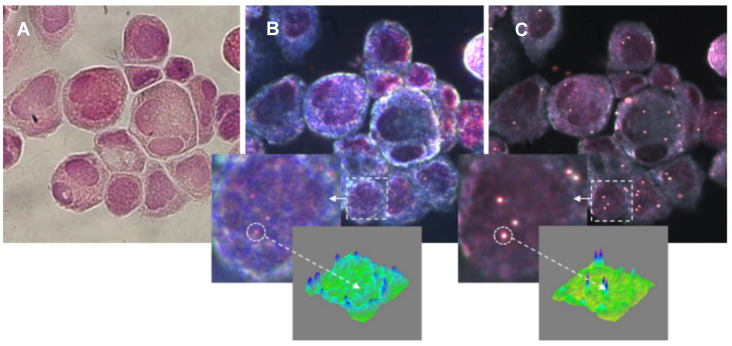
Comparison of the (**A**) bright-field mode, (**B**) transmission based dark-field mode and (**C**) dark-field SI microscopy for the visualization of H&E-stained cytology sample labeled with gold spherical NPs (100 nm in diameter). The inset shows 2D and 3D NP intensity profiles for the contrast comparison. Note that the sample is observed by simply switching from one mode to another.

Compared to standard transmission dark-field microscopy, the SI optical system can improve the contrast between NPs and cells (which scatter light strongly in the forward direction) using an LED-based light source perpendicular to the optical axis (Figure 1). The results of this comparison of the bright-field mode, the transmission-based dark-field mode, and the SI dark-field mode for visualizing the H&E-stained cytological sample labeled with gold spherical NPs (100 nm in diameter) are shown in Figure 2.

**Figure 3:**
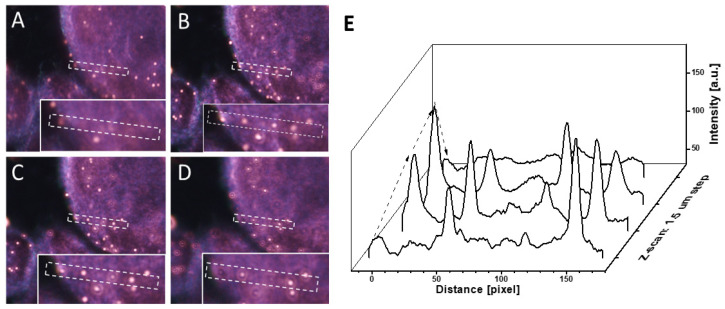
Z-scan of H&E-stained cytology sample labeled with gold spherical NPs (100 nm in diameter). (**A**–**D**) Images were taken with Nikon 60× objective (0.7 NA) at 1µm step. (**E**) Intensity profile of NPs at different Z focal planes.

Another advantage of side illumination is that there are no limitations on the numerical aperture of the imaging objective that exists in transmission dark-field microscopy. The use of a higher NA provides improved image resolution in the Z-direction, which leads to the possibility of 3D mapping of the spatial position of plasmonic markers. Figure 3 presents the image of a H&E-stained specimen labeled with gold spherical NPs (100 nm in diameter) at different focal points with a Z step of 1 μm. By following the position of the peak of the NPs intensity profile, the exact spatial position of each individual NPs on the cell membrane can be obtained.

**Figure 4:**
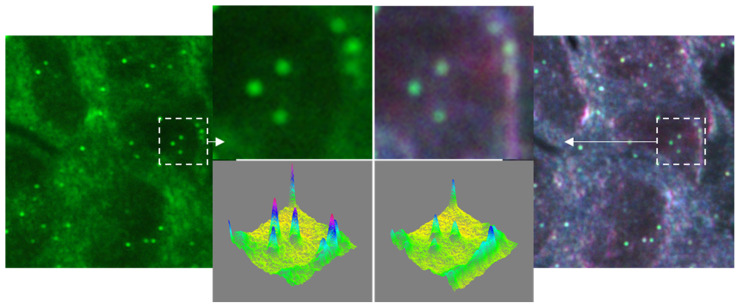
Monochromatic (green LED) and RGB SI dark-field imaging H&E-stained cytology sample labeled with alloy spherical NPs (74 nm in diameter). Images were taken with Nikon 60× objective (0.7 NA). Insets compare the intensity profile of the NPs.

The individual NP-cells contrast, and the resulting NP detection ability also depend on the spectral properties of the NP, as well as on the spectral range of the used light source. In Figure 4, we present the dark-field SI images of a H&E-stained cytological specimen labeled with spherical alloy NPs (74 nm in diameter) and illuminated, respectively, with a green LED and a complete RGB light. Since the spectral range of the green LED is close to the peak of the plasmon spectrum of the alloy nanoparticles, we expect an improvement in the image contrast of the NPs. Accordingly, we confirm this phenomenon and propose a methodology for improving NPs detection, especially in automatic imaging mode, in which the LED color illumination is modulated depending on the type of plasmonic marker.

### 3.2. Plasmonic Bioimaging of Cytology Samples

H&E stain is commonly used by pathologists and researchers for investigating underlying cellular and tissue structures. However, almost all special staining and detection approaches combined with the H&E staining protocol have failed to provide complementary and conclusive information [9,10,11]. To evaluate the performance of our multiplex plasmon biomarkers for cytopathological diagnosis, and in combination with the conventional H&E staining approach, four different types of plasmonic NPs were selected. Their physicochemical characteristics and corresponding spectral properties were theoretically predicted for reliable differentiation, even with three spectral lines (468 nm, 526 nm, and 638 nm) of SI adaptor LED RGB-illumination (Table 1).

The H&E stain is quite intense, and it is not at all obvious that any optically active biomarkers can be reliably visualized and detected. In the case of plasmonic NPs, the detection of extinction/scattering spectra against the diffusion background of H&E stain is also problematic and requires verification. To solve this problem and to verify the effect of H&E staining (Gill II or Harris hematoxylin and eosin B or Y) on the visualization of individual and multiplexed plasmonic NPs, two types of prepared samples (non-embedded and FFPE breast MDA-MB-453 cancer cells) were evaluated. Sample preparation of MDA-MB-453 cells included an incubation for one hour with plasmonic NPs, and then H&E staining prior to visualization under SI dark field microscopy.

**Figure 5:**
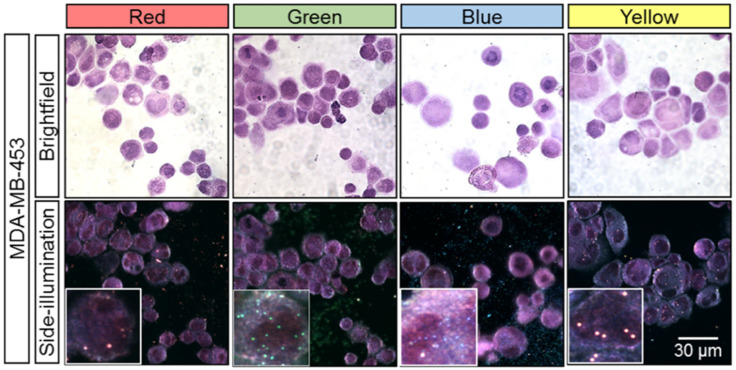
Adherent MDA-MB-453 cells stained with H&E (Gill II hematoxylin and eosin B) and observed with either bright-field or dark-field microscopy by using the SI microscopy adaptor for visualizing red, green, blue, and yellow plasmonic NPs. Insets: 15 × 15 µm.

Our preliminary microscopic results showed that the use of eosin Y to stain fixed adherent cells (non-embedded) strongly influenced the detection performance of plasmonic NPs in this complex system. In this study, a strong green scattering background was observed under SI microscopy. Plasmonic NPs were still visible and distinguishable using digital imaging, but the contrast, in our opinion, was insufficient for reliable visual control. In Figure 5, we present the results for adherent MDA-MB-453 cells stained with H&E (hematoxylin Gill II and eosin B) and observed by bright- or dark-field microscopy using an SI microscopy adapter to visualize red, green, blue, and yellow plasmonic NPs. The bright spots of different colors related to the non-specific cell binding plasmonic NPs that clearly appeared on the delimited cells (obvious and distinguishable boundaries) in the SI images and did not interfere with the H&E bright-field images (morphological context).

**Figure 6:**
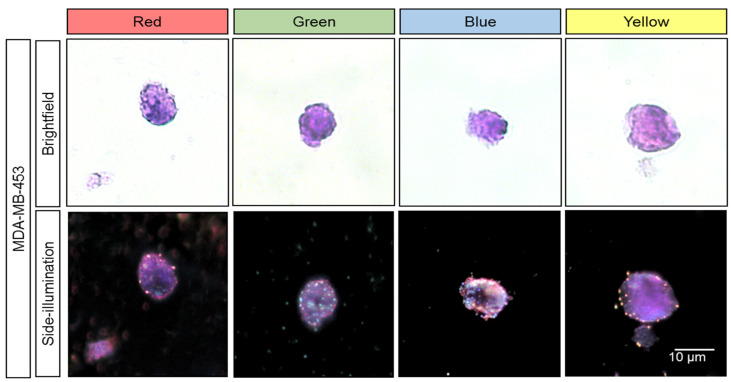
FFPE cells (MDA-MB-453) with H&E staining (Gill II hematoxylin and eosin B) observed with either bright-field or dark-field microscopy by using SI microscopy adaptor for visualizing red, green, blue, and yellow plasmonic NPs.

In pathology’s daily practices, FFPE samples are commonly prepared for cancer diagnosis, and therefore it is critical to evaluate the use of plasmonic NPs in combination with H&E (Gill II hematoxylin and eosin B) stained FFPE samples. As shown in Figure 6, FFPE MDA-MB-453 cells were incubated with the same four types of plasmonic NPs, H&E stained and visualized under SI microscopy. The images indicate no color fading or other interferences that may lead to misinterpretations. All plasmonic NPs on the treated cells were clearly observed under the exact same illumination and camera settings without interfering other staining protocols.

**Figure 7:**
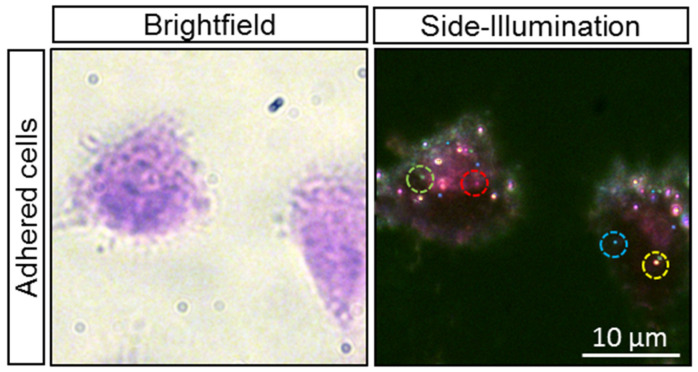
The representative bright-field and dark-field SI images of multiplexed MDA-MB-453 adherent cells stained with Gill II hematoxylin and eosin B.

To evaluate the potential of the plasmonic bioimaging in a multiplexed detection system combined with the H&E-stained samples, a mixture of four different fresh plasmonic NPs has been incubated with the samples (Gill II hematoxylin and eosin B) before the H&E staining steps and visualized using bright-field and SI microscopy (Figure 7). The bright-field images show the morphology of the H&E-stained cells, whereas the dark-field SI images clearly illustrate four different plasmonic NPs. Note that the observation of plasmonic NPs in stained 3D cells strongly depends on the focal plane. Plasmonic NPs that are out of focus can appear with a lower contrast and even a different color due to local interference effects. However, with manual or automatic Z-scans, pathologists and experienced operators can visually distinguish different colored NPs using the correct focal plane. Similar results were obtained for FFPE samples (not shown), which confirms the possibility of biomarkers multiplexing in the proposed approach.

## 4. Discussion

The H&E counterstaining in the standard cytopathology techniques is central to provide accurate morphological diagnosis [7]. The strong background of H&E counterstaining was the main reason for the complication of integration with other diagnostic approaches and optical biomarkers. However, as we presented in this article, plasmonic NPs with their stable and strong resonant light scattering ability have been proven as compatible biomarkers with the H&E stain procedure. Four types of spectrally distinctive plasmonic NPs have been clearly observed on either FFPE (thin layer ~4 µm) or non-embedded cells without suppressing the performance of standard morphological diagnosis. Additionally, the four distinctive markers added to the cells demonstrated the potential ability of multiplexed plasmonic labeling to specifically diagnose cancer cells with two positive markers, and two negative markers placed in cytological samples.

A major challenge in integrating new approaches, including the presented plasmonic bioimaging, with standard cytopathology protocols, is the resistance to these technologies from hospitals and medical personnel. The introduction of new technologies can be facilitated if they demonstrate their effectiveness in direct comparison with existing methods, generating additional relevant information about the same cytopathological samples and within the same analysis procedure. To meet this requirement, as shown in this article, the dark-field side-illumination adaptor was specifically designed to be easily integrated into a conventional microscope commonly used by a pathologist. The SI adaptor does not interfere with the standard diagnostic protocol and is used to detect plasmonic NP markers on cells stained with H&E (hematoxylin Gill II and eosin B).

Moreover, a sensitive and precise detection of cell receptors and proteins needs to be adapted into clinical settings using functionalized plasmonic NPs in this state-of-the-art visualization approach. In future works, several robust functionalization methods [20,21,22,23], which have already been confirmed as a reliable and selective biomarker conjugation for recognizing cell receptors and proteins, will be optimized and applied on plasmonic NPs with respect to standard cytological staining protocols in order to provide multispectral imaging and multiplex immunolabeling, and facilitate a quantitative examination in the existing standard pathology workflow.

## 5. Conclusions

A promising integrated bioimaging approach has been obtained by taking advantage of the optical properties of plasmonic NPs as complementary biomarkers for improving the performance of standard cytopathology techniques. For the first time, the successful combination of the plasmonic bioimaging with the stained FFPE samples has been demonstrated, opening the potential introduction of a simple and quantitative cancer diagnostic technique for clinics and hospitals. This established integration of plasmonic bioimaging on H&E-stained cytology samples as a proof of principle facilitates an engineering-driven workflow for standardizing immunoplasmonic-based cancer diagnosis. Given the high incidence of cancer, the development of an adaptable, sensitive, and specific diagnostic protocol based on plasmonic NPs can provide an indispensable tool for improving cancer diagnosis and daily care.

## Figures and Tables

**Table 1:** Characterization of plasmonic NPs with different resonance peaks.

Color	Geometry	Composition	Size (nm)	Plasmon Peak (nm)
Blue	Sphere	Gold/Silver Alloy 10/90% vol.	43	457
Green		Gold/Silver Alloy 50/50% vol.	74	529
Yellow		Gold	100	607
Red	Rod	Gold	25 × 71	669

## Data Availability

Not applicable.
